# Photosensitive Layer-by-Layer Assemblies Containing Azobenzene Groups: Synthesis and Biomedical Applications

**DOI:** 10.3390/polym9110553

**Published:** 2017-10-25

**Authors:** Uichi Akiba, Daichi Minaki, Jun-ichi Anzai

**Affiliations:** 1 Graduate School of Engineering and Science, Akita University, 1-1 Tegata Gakuen-machi, Akita 010-8502, Japan; uakiba@gipc.akita-u.ac.jp; 2 Graduate School of Pharmaceutical Sciences, Tohoku University, Aramaki, Aoba-ku, Sendai 980-8578, Japan; daichi.minaki.e3@tohoku.ac.jp

**Keywords:** azobenzene, photoresponse, layer-by-layer, thin film, microcapsule, photosensitive, cell adhesion, ion gate, controlled release

## Abstract

This review provides an overview of the syntheses of photosensitive layer-by-layer (LbL) films and microcapsules modified with azobenzene derivatives and their biomedical applications. Photosensitive LbL films and microcapsules can be prepared by alternate deposition of azobenzene-bearing polymers and counter polymers on the surface of flat substrates and microparticles, respectively. Azobenzene residues in the films and microcapsules exhibit trans-to-cis photoisomerization under UV light, which causes changes in the physical or chemical properties of the LbL assemblies. Therefore, azobenzene-functionalized LbL films and microcapsules have been used for the construction of photosensitive biomedical devices. For instance, cell adhesion on the surface of a solid can be controlled by UV light irradiation by coating the surface with azobenzene-containing LbL films. In another example, the ion permeability of porous materials coated with LbL films can be regulated by UV light irradiation. Furthermore, azobenzene-containing LbL films and microcapsules have been used as carriers for drug delivery systems sensitive to light. UV light irradiation triggers permeability changes in the LbL films and/or decomposition of the microcapsules, which results in the release of encapsulated drugs and proteins.

## 1. Introduction

**Figure 1:**
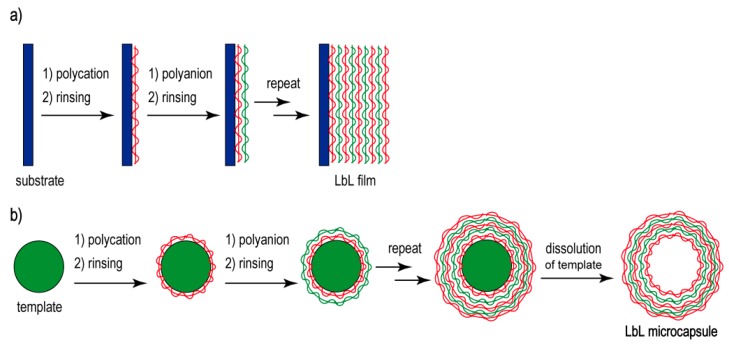
Preparation of layer-by-layer (LbL) films (**a**) and microcapsules (**b**).

Layer-by-layer (LbL) deposition of polymeric materials on the surface of a solid substrate creates multilayer thin films as a layered structure. Polymeric materials are linked to each other in the films through attractive forces including electrostatic interactions, hydrogen bonds, covalent bonds, molecular recognition, and biological affinity. LbL-deposited films were first prepared in the early 1990s by the alternate deposition of cationic and anionic polymers through electrostatic interactions [1,2,3]. Since then, a variety of synthetic and biological materials have been used as components of LbL films. Biopolymers such as proteins [4,5], polysaccharides [6,7], and DNA [8,9] are often used as the building blocks of LbL films because these biopolymers contain electrical charges. A merit of the LbL deposition technique is that films can be deposited on a variety of solid substrates including metals, glass, ceramics, and biological samples such as living cells. In a typical procedure, the solid substrate is alternately immersed in aqueous polymer solution for 15–30 min to deposit polymers on the surface of the substrate, followed by rinsing to remove weakly adsorbed polymers (Figure 1a). The thickness of the films can be regulated simply by changing the number of deposited layers because film thickness increases with the number of depositions.

Hydrogen bonding is also available as a driving force for the preparation of LbL films. Poly(acrylic acid) (PAA) and poly(methacrylic acid) (PMA) are often used to construct hydrogen bond-based LbL films by combining hydrogen bonding acceptors such as poly(ethyleneglycol) and poly(vinylpyrrolidone) [10,11]. LbL films consisting of PAA or PMA are stable in acidic media. However, the hydrogen bond-based LbL films are unstable in solutions of neutral and basic pH because the hydrogen bonds are broken as a result of deprotonation of the carboxylic acid residues. Therefore, hydrogen-bonded LbL films have been widely used for the preparation of pH-sensitive devices [12,13,14]. 

Binding proteins such as concanavalin A (Con A) [15,16] and avidin [17,18] are employed as protein materials for the construction of LbL films. Con A and avidin are known to selectively bind sugars and biotin. Thus, sugar- and biotin-labeled materials can be built into LbL films by using Con A and avidin. For instance, glycoenzymes equipped with intrinsic hydrocarbon chains, such as horseradish peroxidase [19] and glucose oxidase [20], can be used as film components without labeling in the Con A-based protocol. Interestingly, the catalytic activity of the enzymes is retained even in the LbL films. 

Other molecular interactions such as charge-transfer (CT) interactions [21], DNA hybridization [22], host-guest complexations [23], π-cation interactions [24], coordination bonds [25], and covalent bonds [26] are also available as binding forces for the construction of LbL films. These molecular interactions are characterized by the high selectivity in binding. For example, nucleotide chains with complementary base pairs can be hybridized to form LbL films [22]. Similarly, only the guest compounds with suitable shape and size are included in the cavity of host molecules in the films [23]. In addition, specifically designed polymers have to be synthesized to construct LbL films based on these molecular interactions. In fact, carbazole and dinitrophenyl groups as CT donor and acceptor, respectively, are introduced in the side chain of polymers for constructing CT-based LbL films [21]. In another case, a combination of pyridine-bearing materials and ruthenium complexes was employed in LbL films prepared through coordination bonds [25].

LbL films can be used to construct hollow microcapsules [27]. To achieve this, LbL films are deposited on the surfaces of colloidal particles, followed by dissolution of the core (Figure 1b). Inorganic microparticles such as CaCO_3_ are often used to encapsulate proteins and drugs in microcapsules because the template can be dissolved in mild aqueous media [28,29]. The permeability of microcapsules thus prepared can be manipulated by changing the environmental pH, which enables the LbL microcapsules to be used as drug carriers. In addition, the structure of their shell membranes can be tuned at the molecular level by using suitable building block materials. Thus, LbL films and microcapsules are currently utilized in a variety of devices, including edible coatings for foods [30], filtration membranes [31], biosensors [32,33], and drug delivery systems [34,35].

**Figure 2:**
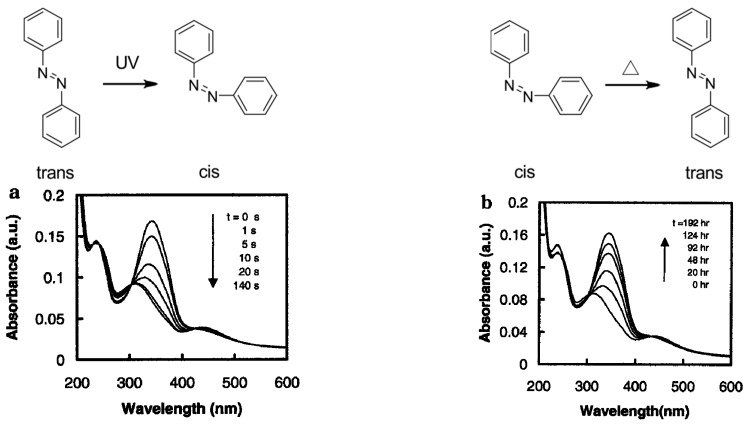
Typical UV–visible absorption spectra of an azobenzene-containing LbL film for trans-to-cis isomerization under UV light (**a**) and those of thermal cis-to-trans isomerization in the dark (**b**). Reprinted with permission from Ref. [50]. Copyright 2001 American Chemical Society.

Recently, much attention has been devoted to LbL assemblies that change their physical or chemical properties in response to external stimuli [36,37,38]. Photoirradiation is a typical stimulus that can induce changes in the structures and properties of organic thin films. A variety of photosensitive compounds have been used as photoreceptors to construct photosensitive devices [39,40,41,42,43]. Among them, azobenzene derivatives have been most widely used owing to their reasonable stability in UV and visible light. Azobenzene derivatives are known to exhibit a characteristic absorption band in the UV–visible absorption spectra which originates from the π–π* transition of the azobenzene chromophore. The absorption intensity and wavelength of the spectra depends significantly on the conditions of the solution, such as pH. In addition, azobenzene derivatives undergo reversible trans–cis isomerization under photoirradiation, resulting in significant changes in the molecular geometry, absorption spectra, redox properties, and so forth [44,45,46]. Early works demonstrated that azobenzene residues exhibit trans–cis isomerization even in LbL films upon UV and visible light irradiation [47,48,49,50]. Figure 2 shows the typical UV–visible absorption spectra of an azobenzene-containing LbL film before and after UV light irradiation, together with conformational changes of the azobenzene moiety. The strong absorption band in the UV spectrum before irradiation originates from the π–π* transition of the trans form of the azobenzene chromophore, while the weaker absorption band at longer wavelengths is ascribed to n–π* transition. The cis isomer is metastable and will undergo thermal isomerization back to the trans form in the dark, or photochemical isomerization under visible light. The intensity of the π–π* transition band can be used to estimate the amount of trans isomers in the irradiated samples, under the assumption that the absorption of the cis isomer is negligible at the given wavelength. Thus, it is reasonable to assume that the macroscopic properties of azobenzene-containing LbL films and microcapsules are altered upon photoirradiation. 

This review focuses on the synthesis of LbL films and microcapsules containing azobenzene-modified polymers and their biomedical applications. We discuss the structure and properties of azobenzene-containing LbL assemblies in relation to the photoinduced trans–cis isomerization of the azobenzene moieties in the assemblies.

## 2. Synthesis of Azobenzene-Containing LbL Assemblies

Azobenzene-containing LbL films and microcapsules can be constructed using different strategies. The first strategy uses azobenzene-modified polymers as components of the films and microcapsules. In this method, azobenzene-modified polymers are deposited on a solid surface in combination with a counter polymer. The second strategy relies on the post-modification of prepared LbL assemblies with azobenzene derivatives through covalent bonding. This strategy provides a simple way to construct azobenzene-containing LbL assemblies. However, it can be difficult to maintain precise control of the azobenzene content in the LbL assemblies. Therefore, in most cases, azobenzene-containing LbL assemblies are constructed through the first strategy.

**Figure 3:**
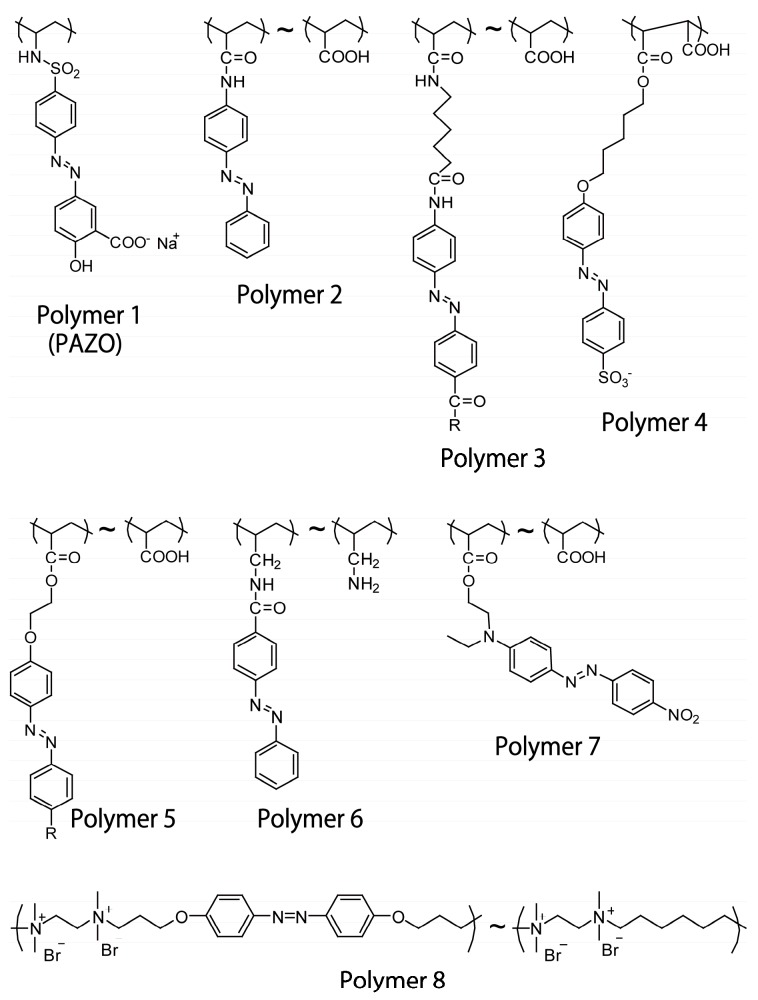
Chemical structures of azobenzene-modified polymers used for the construction of LbL films and microcapsules.

**Figure 4:**
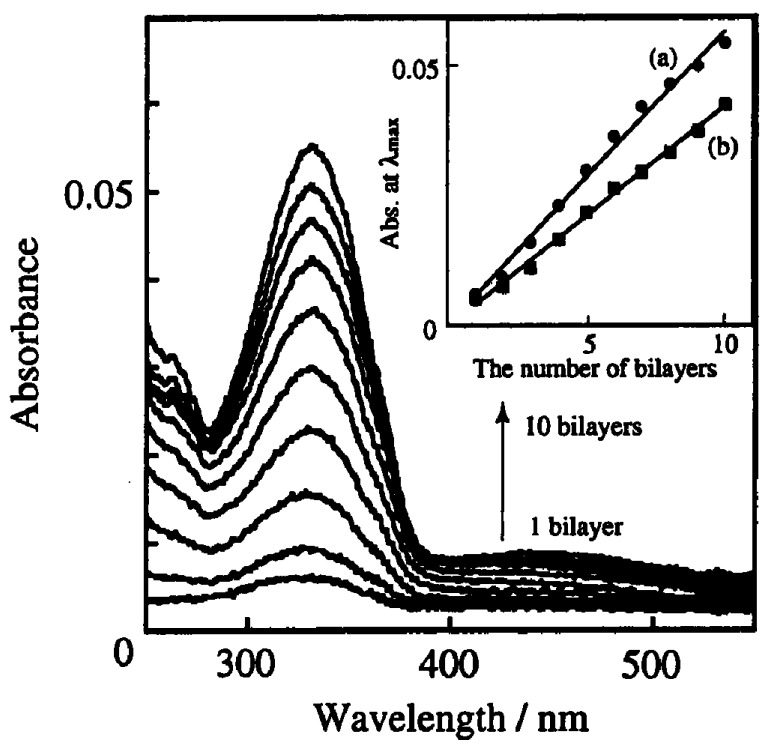
UV–visible absorption spectra of LbL films composed of Polymer **6** and PSS as a function of the number of layers. Inset shows the plots of absorbance of the Polymer **6**/PSS (a) and Polymer **6**/PVS (b) films at 330 nm. Reprinted with permission from Ref. [57]. Copyright 2002 American Chemical Society.

The chemical structures of typical azobenzene-containing polymers used as components of LbL films are shown in Figure 3. The polymers comprise azobenzene moieties in their side chains or backbone. Among them, poly[1-[4-(3-carboxy-4-hydroxyphenylazo)-benzenesulfonamide]-1,2-ethanediyl sodium salt] (Polymer **1**) is widely used as an anionic component in LbL films because it is commercially available from Sigma-Aldrich (St. Louis, MO, USA) (Polymer **1** is often referred as PAZO). PAZO can be built into LbL films by alternate deposition with cationic polymers such as poly(allylamine hydrochloride) (PAH), poly(ethyleneimine), and poly(diallyldimethylammonium chloride) (PDDA) [47,48,51,52,53]. PAZO LbL films show high stability in water and in a range of organic solvents. PAZO exhibits trans-to-cis isomerization in the films upon UV light irradiation. Polymers **2** and **3** are not commercial products but can be synthesized by polymerization of the corresponding monomers [54,55]. Alternatively, post-modification of parent polymers through amide and ester linkages (Polymers **4**–**6**) [50,56,57] or diazo-coupling reaction (Polymer **7**) [58,59,60] are available for the construction of azobenzene polymers. Polymer **6** has been used to construct LbL films by combining polyanions such as poly(vinyl sulfate) (PVS) and poly(styrene sulfonate) (PSS) [57], while Polymers **2**–**5** and **7** have been used as anionic components in LbL films. The contents of azobenzene moieties in the polymers can be easily regulated by changing the amounts of azobenzene residues during synthesis. Thus, it is possible to regulate the physical and chemical properties of LbL films. Figure 4 shows UV–visible absorption spectra of LbL films consisting of Polymer **6** and PSS or PVS [57]. The π–π* transition band at 330 nm of the azobenzene residues in Polymer **6** linearly increased as the number of depositions increased, showing that the same amount of Polymer **6** is immobilized in each layer of the LbL films. The loading of azobenzene residues in each layer was estimated to be (0.9 − 1.2) × 10^−6^ g cm^−2^ in the LbL films, based on the intensity of the absorption band of azobenzene residues at 330 nm. Thus, the deposition behavior of LbL films containing azobenzene polymers can be monitored by recording the UV–visible spectra.

Polymers with main-chain azobenzene groups are also available for the construction of LbL films (Polymer **8**) [61,62,63,64]. These polymers are characterized by flexible alkylene chains linked with rigid azobenzene moieties. Interestingly, LbL films prepared using the cis isomers of polymers provide thicker layers than those prepared from trans isomers, probably due to the coiled conformation of the cis polymers in contrast to the stretched conformation of the trans form [61,62]. Increasing the spacer length of Polymer **8** creates LbL films with dense packing of the azobenzene groups resulting from the strong aggregation of the azobenzene residues [64].

## 3. Biomedical Applications of Azobenzene-Containing LbL Assemblies

### 3.1. Photo-Controlled Cell Adhesion

A key issue in the development of biomedical devices, such as implantable organs and biosensors, is the adhesion of cells to solid surfaces. It is widely recognized that cell adhesion is affected by several factors including the amount of electrical charges, stiffness, and the roughness of the surfaces. In general, negatively charged surfaces are expected to exert electrostatic repulsion effects on mammalian cells because the surfaces of the cells often contain excess negative charges originating from anionic species such as carboxylate and phosphorylate residues [65]. Cells are adsorbed more strongly onto the surfaces of stiff substrates than onto soft surfaces [66]. Thus, cell adhesion can be regulated by changing these factors through changes in external stimuli such as pH, temperature, electric potential, and light [67,68,69,70]. In fact, LbL films have been utilized to manipulate surface properties to promote the adhesion of mammalian cells to the surfaces while preventing the adhesion of bacterial cells [70].

**Figure 5:**
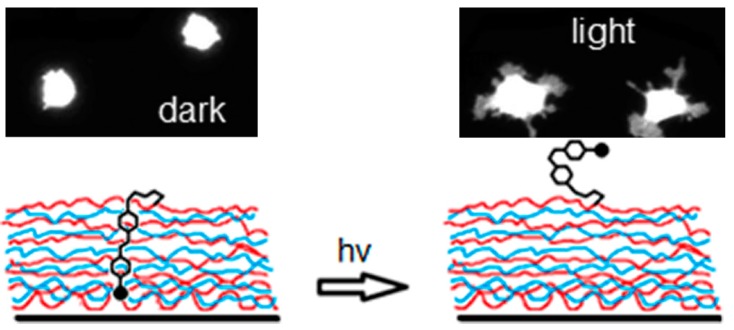
Typical cell surface area on the LbL films in darkness and under UV–light irradiation (**top**) and photo-induced conformational change of RGD-attached azobenzene residues in the LbL film (**bottom**). Reprinted with permission from Ref. [54]. Copyright 2012 American Chemical Society.

In this context, azobenzene-containing LbL films have been employed to regulate cell adhesion by photoirradiation. Barrett and coworkers have prepared LbL films composed of Polymer **3**, in which the azobenzene group is tethered with a tripeptide ligand consisting of arginine, glycine and aspartic acid (RGD) [54]. The RGD sequence is known to be an essential motif found in cell-adhesive proteins such as fibronectin, which binds to the integrin family of cellular transmembrane proteins [71]. A small fraction (less than 1%) of the azobenzene residues in the LbL films exhibit trans-to-cis photoisomerization upon UV light irradiation, exposing the azobenzene moieties at the surface (Figure 4). The photo-induced positioning changes result in a 40% enhancement in adhesion and the survival of NIH 3T3 cells on the LbL films (Figure 5). LbL films composed of Polymer **7** have been used to promote the adhesion and growth of cerebellar neurons from rat pups [72]. Apart from LbL films, RGD tripeptide-modified azobenzene derivatives have been used to construct self-assembled monomolecular (SAM) layers on solid surfaces to control cell adhesion [73]. These azobenzene-containing LbL films as well as SAM may be useful for future studies of the photo-induced construction of neural pathways and cell arrays. 

Photo-switched cell adhesion on the surfaces of LbL films has been compared with that on chemisorbed azobenzene films [74]. The LbL film and the chemisorbed film were functionalized with identical 3-trifluoromethyazobenzene residues. The UV–visible absorption spectra of the films showed that UV light irradiation yielded a photo-stationary state with 8.3% of cis isomers in the chemisorbed film. However, the LbL film contained 48.4% cis isomers at the photo-stationary state due to the loose packing of the azobenzene residues. Trans-to-cis isomerization of the azobenzene groups in the LbL film induced a significant change in the wettability of the surface: the contact angle decreased from 141.4° to 110.3° upon UV light irradiation. The number of breast (MCF-7) and bladder (T24) cancer cells adsorbed on to the UV light-irradiated LbL film was significantly decreased, probably due to the increased wettability of the irradiated film. Thus, this study demonstrated the superiority of azobenzene-containing LbL films over chemisorbed films for the regulation of cell adhesion.

### 3.2. Photo-Controlled Ion Gating

**Figure 6:**
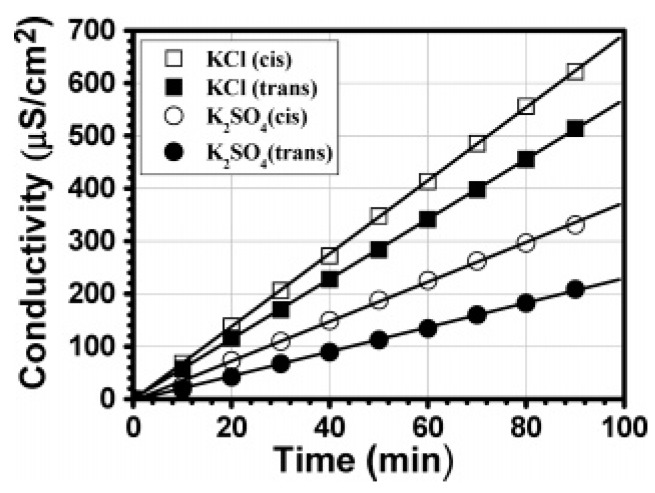
Ion permeability of porous alumina membranes coated with LbL films under UV light (open circles and squares) and in the dark (filled circles and squares) measured by conductivity. Reprinted with permission from Ref. [75]. Copyright 2008 American Chemical Society.

Changes in ion permeability or ion gating effects in biological membranes form the basis of signal transduction across the membranes. Therefore, the development of photo-switched ion gating systems is of interest for future biomedical applications. Hong and coworkers studied the photosensitive ion permeability of porous alumina membranes coated with azobenzene-modified LbL films [75]. Figure 6 shows the increase in conductivity of the receiving-phase solutions of the permeation cell, which demonstrated accelerated permeation of KCl and K_2_SO_4_ across LbL film comprising cis azobenzene. The permeation rate of the Cl^−^ ion increased from 6.40 × 10^−7^ to 7.74 × 10^−7^ cm s^−1^ and that of the SO_4_^2−^ ion from 2.66 × 10^−7^ to 4.06 × 10^−7^ cm s^−1^. The ion-gating effects were reversible several times. The results were rationalized based on the enhanced sizes of the ion channels alongside a minor effect of greater swelling in the UV light-irradiated LbL film. 

**Figure 7:**
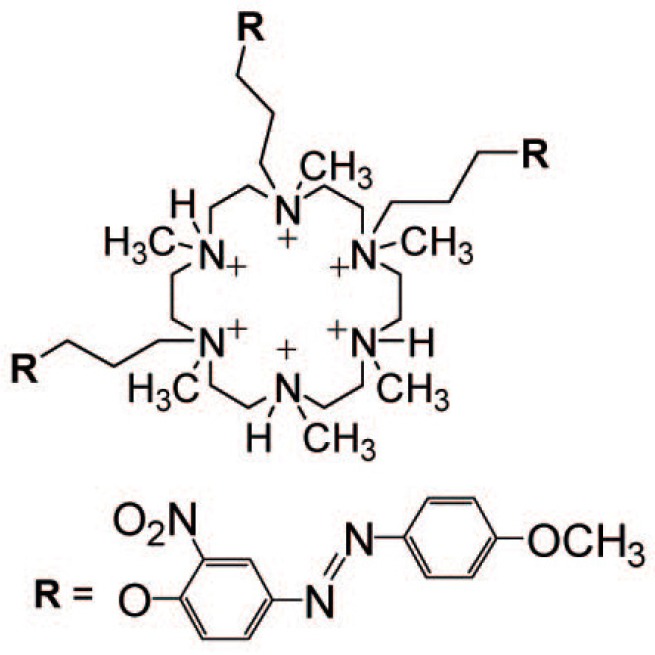
Chemical structure of azobenzene-tethered macrocyclic azacrown ether. Reprinted with permission from Ref. [76]. Copyright 2009 American Chemical Society.

Azobenzene-tethered macrocyclic azacrown ether, 1,4,10-[3-(4-(4′-methoxyphenylazo)-2-nitrophenoxy)propyl]-1,4,10,13,16-hexamethylhhexaazacyclooctadecane (Figure 7), has been used as a cationic component of LbL films to control ion permeability across LbL film-coated porous alumina membranes [76]. The ion permeation rates of the LbL film were 7.89 × 10^−7^ cm s^−1^ for the Cl^−^ ion and 5.93 × 10^−7^ cm s^−1^ for the SO_4_^2−^ ion before irradiation, while the values were enhanced to 8.04 × 10^−7^ cm s^−1^ and 6.28 × 10^−7^ cm s^−1^, respectively, after UV light irradiation. Thus, the ion permeation across the LbL film was accelerated by UV light irradiation. The authors reported that ion permeability is enhanced by changes in the pore sizes of the LbL film, which in turn originates from the different orientation of trans and cis azobenzene chromophores in the film. These results suggest that azobenzene-containing LbL films are promising materials for the construction of photo-sensitive ion gates. However, the effect of photoirradiation is still limited: the permeation rates of the irradiated LbL film for the Cl^−^ and SO_4_^2−^ ions are only 1.02 and 1.06 times higher than those before irradiation. Further improvements may be required for the application of these systems in biological fields.

High-performance LbL films with appropriate ion permeability are required for the development of photosensitive ion gates. In this context, an interesting protocol has been developed based on the effects of a high gravity field. Shi and coworkers deposited PAZO and PDDA on solid surfaces under a high-gravity field using homemade high-gravity equipment with a rotator, and the ion permeability and photoresponses of the films were compared with LbL films prepared by a conventional dipping procedure [77]. Cyclic voltammetric studies on the LbL films showed that the LbL films prepared under the high-gravity field had lower ion permeability than the conventional films, which indicated compact packing of PAZO and PDDA. In addition, the rate of photoisomerization of PAZO was slower in the new films than in conventional films, probably owing to the limited free volume in compact films. The ion permeability and photoresponses of the PAZO/PDDA films are adjustable by controlling the magnitude of the gravity field. 

### 3.3. Photo-Controlled Release

LbL films and microcapsules have been applied to the construction of controlled-release systems [78,79,80,81]. Functional molecules such as drugs and proteins can be embedded in LbL films and microcapsules as a film component during construction, or as an additive in prepared LbL films and microcapsules. The first strategy allows precise regulation of the amounts of the drugs and proteins in the films by changing the number of LbL layers, whereas precise control is difficult to achieve via the second route. Microcapsule shell membranes are usually highly permeable to drugs and proteins. A third route for encapsulation is to use porous particles preloaded with proteins or drugs. In this method, the template particles are coated with LbL films, and then the template dissolves away leaving the drugs or proteins in the microcapsules. The encapsulation efficiency of this method is high.

**Figure 8:**
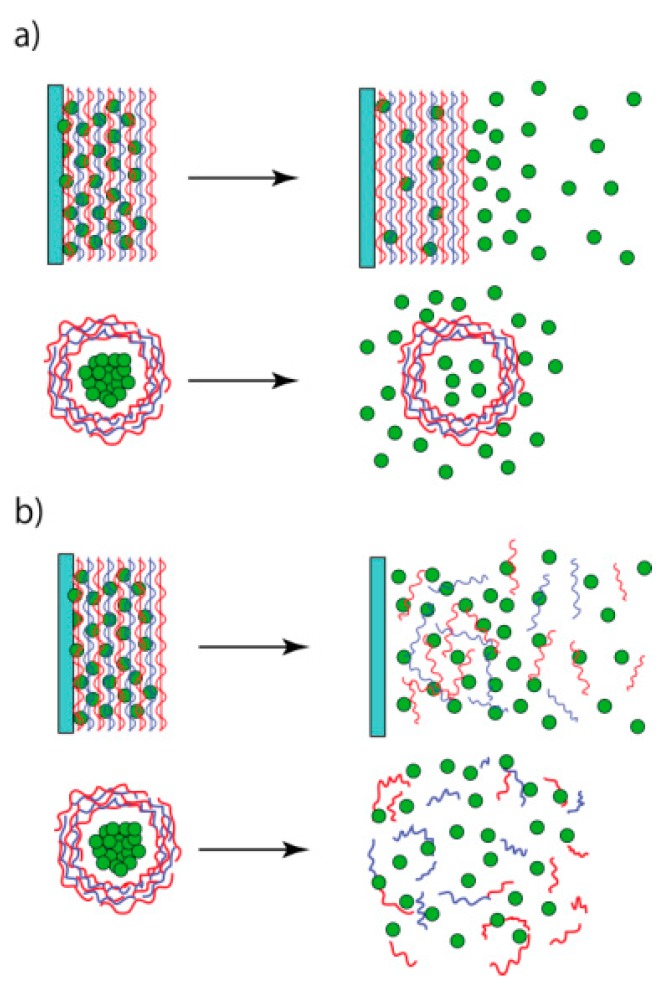
Release of drugs and proteins from LbL films and microcapsules through permeability changes (**a**) and decomposition (**b**). Reprinted from Ref. [82]. Copyright 2017 MDPI.

The loaded materials are released from LbL films and microcapsules upon exposure to external stimuli that increase the permeability of the film or cause the film to decompose (Figure 8) [82]. Consequently, a key issue in the development of stimuli-sensitive release systems is the design of suitable stimuli-sensitive materials for the LbL assemblies. A variety of stimuli have been employed to trigger release, including changes to the pH, temperature, electrical fields, and the concentration of ions and small molecules [36,37,38,83,84]. In this section, we discuss examples of azobenzene-based LbL films and microcapsules for controlled release.

**Figure 9:**
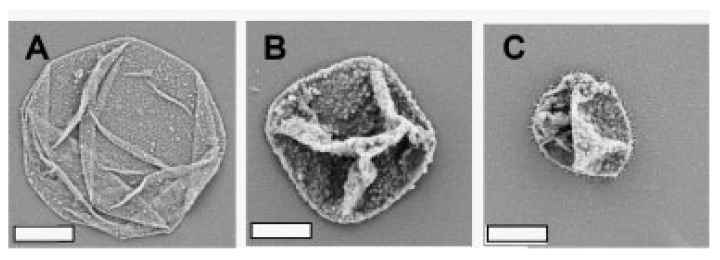
Scanning electron microscopy images of dried PAZO/PDDA microcapsules before irradiation (**A**), after 60 min (**B**) and after 8 h of UV light irradiation (**C**). The scale bars represent 2 μm. Reprinted with permission from Ref. [85]. Copyright 2007 WILEY-VCH.

The permeability of azobenzene-containing LbL films and microcapsules can be altered by photoisomerization of the azobenzene residues. Sukhorukov and coworkers have reported the photoinduced shrinkage of microcapsules made of PAZO and PAH [85]. Figure 9 shows scanning electron microscopy images of the PAZO/PAH microcapsules before and after UV light irradiation. Because of the shrinkage, the PAZO/PAH microcapsules firmly encapsulated a fluorescent-labeled dextran after UV light irradiation, while labeled dextran freely leaked from the non-irradiated capsules. The results were rationalized based on changes in the permeability of the microcapsule shell. On the other hand, LbL microcapsules consisting of PAZO and PDDA exhibit different types of photoresponses. PAZO/PDDA microcapsules decompose under UV light irradiation, which is caused by the photoinduced rearrangement of PAZO in the capsule shell [86]. UV–visible absorption spectra of the microcapsules indicate the formation of head-to-tail aggregates of azobenzene residues (i.e., J-aggregates) in the capsule shell. The release of bovine serum albumin from the PAZO/PDDA microcapsules is highly accelerated under UV light irradiation owing to the decomposition of the capsules.

**Figure 10:**
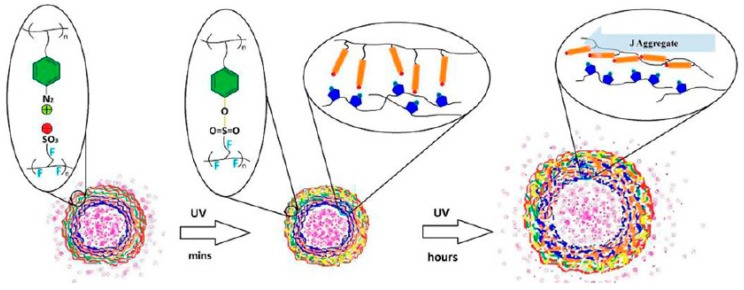
Photoresponses of dual-functional LbL microcapsules composed of (PAZO/PDDA) + (diazo resin (DAR)/Nafion) shells. Reprinted with permission from Ref. [87]. Copyright 2013 American Chemical Society.

The same group further constructed dual-functional LbL microcapsules using composite layers consisting of PAZO/PDDA and diazo resin (DAR)/Nafion layers [87]. DAR is a photosensitive polymer containing a diazonium group in the side chain, which reacts with carbonate and sulfonate groups to form esters under UV light irradiation. In fact, the DAR/Nafion layers underwent rapid cross-linking to form sulfonate esters in the capsule’s shell. Thereafter, prolonged irradiation with UV light induces realignment of the azobenzene residues in the shell, resulting in enhanced swelling of the shell (Figure 10). The encapsulation and release of a fluorescent dye-labeled polysaccharide AF488-dextran (10 kDa) has been studied using LbL microcapsules composed of (PAZO/PDDA) + (DAR/Nafion) shells under UV light irradiation. The results demonstrated greatly enhanced release of AF488-dextran under irradiation compared with that under dark conditions. These results suggest that the photoresponses of composite microcapsules depend on the architecture of the multilayer shells such as the thickness and ratios of the PAZO and DAR layers, as well as the type of counter polyanions. In other words, suitably designed variables would further improve the performance characteristics of the microcapsules. 

**Figure 11:**
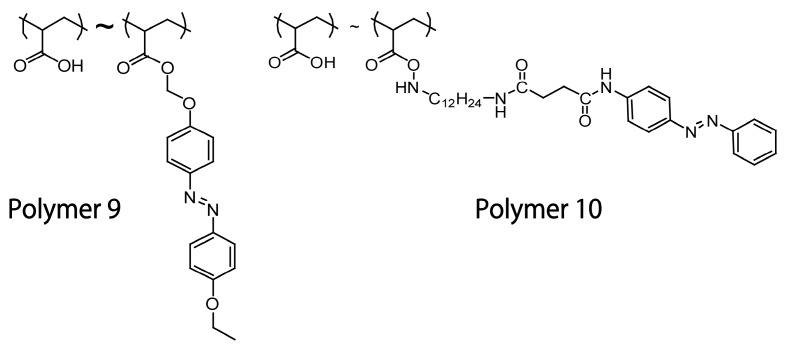
Chemical structures of Polymers **9** and **10**.

**Figure 12:**
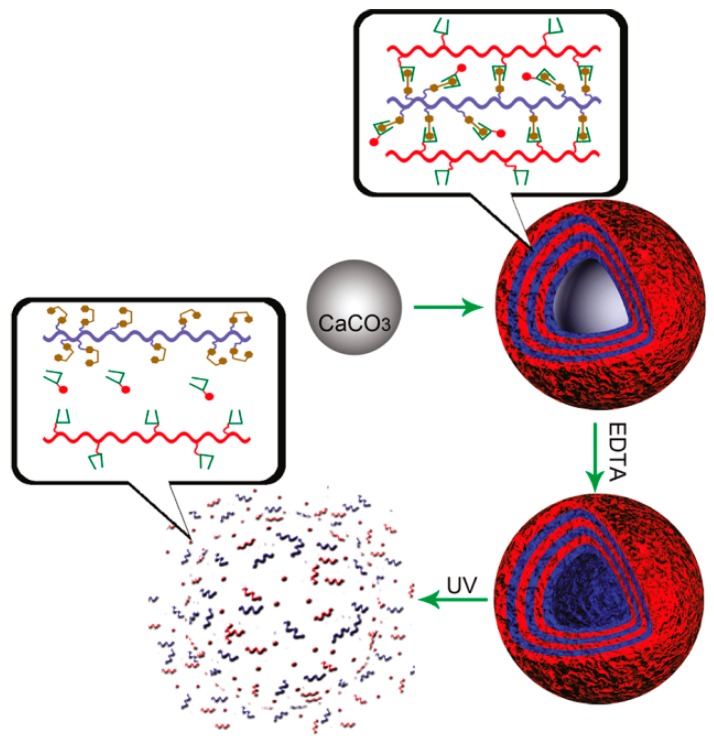
Photo-induced burst release of rhodamine B-labeled α-cyclodextrin (α-CD-RhB) from a LbL microcapsule. Reprinted with permission from Ref. [92]. Copyright 2011 American Chemical Society.

It is known that cyclodextrins (CDs) can accommodate trans azobenzenes in the cavity to form inclusion complexes but they cannot accommodate cis isomers because of their bulkiness. Thus, host–guest complexation between azobenzene derivatives and CDs can be controlled by photoirradiation [88,89,90]. In fact, azobenzene-containing LbL films and microcapsules have been combined with CDs to develop photo-controlled release systems. Zhang and coworkers prepared LbL films composed of azobenzene-modified PAA (Polymer **9**; Figure 11) and PDDA, in which rhodamine B-labeled α-CD (α-CD-RhB) was loaded as a model drug through host–guest complexation [91]. The α-CD-RhB was released from the LbL film upon UV light irradiation due to the trans-to-cis photoisomerization of the azobenzene residues in the film. Visible light irradiation restored the trans-azobenzene isomers and enabled the film to reversibly uptake α-CD-RhB. The loading/release cycle was reversible more than 10 times. LbL microcapsules made of Polymer **10** (Figure 11) and α-CD-modified carboxymethyldextran (α-CD-Dex) have also been used to control the release of α-CD-RhB [92]. The LbL microcapsules decomposed upon UV light irradiation owing to the formation of cis-azobenzene residues in the capsule’s shell, which resulted in a burst release of α-CD-RhB as the model drug (Figure 12). 

It is also possible to construct photosensitive LbL microcapsules that change the permeability without decomposition using a PAA derivative co-modified with azobenzene and adamantane (Azo-Ad-PAA) [93]. LbL microcapsules made of β-CD-modified poly(aspartate) (β-CD-PAsp) and Azo-Ad-PAA do not decompose even under UV light irradiation because of the strong binding of adamantane residues to the β-CD, while photosensitive binding between azobenzene and β-CD can regulate the pore size or permeability of the capsule shell. Notably, the permeability changes of the microcapsules are reversibly regulated by irradiating the microcapsules alternately with UV and visible light.

Theranostic microcapsules, which can be used for magnetic resonance imaging (MRI) and pH-sensitive drug delivery, have been developed using LbL films composed of adamantane-modified poly(aspartate) (Ad-PAsp), azobenzene-modified poly(methacrylate) (Azo-PMA), and β-CD-modified dextran (β-CD-Dex) [55]. The theranostic microcapsules comprised inner Azo-PMA/β-CD-Dex layers and outer Ad-PAsp/β-CD-Dex layers and contained fluorescein-labelled dextran (FITC-Dex) as a model drug. The release of FITC-Dex from the capsules was accelerated by UV light irradiation at low pH. For instance, 37% of the drug was released in the first 2 h under UV light, while 14% was released under dark conditions at pH 6.5. However, at pH 7.4, drug release was only 2% and 1% under UV light and in the dark, respectively. Thus, the effect of UV light is significantly different at a weakly acidic pH. This is because β-CD-Dex contains an acid-sensitive Schiff’s base linker that connects β-CD to dextran chains and can be cleaved at an acidic pH. In vitro cytotoxicity tests performed using HeLa cell lines showed good cytocompatibility. In addition, in vivo acute toxicity tests using mice showed subcutaneous LD_50_ of more than 1000 mg kg^−1^, suggesting that the microcapsules have low toxicity. A potential use of microcapsules loaded with Fe_3_O_4_ for MRI of cancer cells was also suggested from the in vivo studies in mice. 

Multi-drug delivery systems for cancer therapy have been studied using LbL microcapsules prepared from β-CD-PAsp and PAA *co*-modified with azobenzene and adamantane through a proline-leucine-glycine-valine-arginine (PLGVR) peptide linker [94]. The model drugs FITC-Dex and α-CD-RhB were encapsulated in the cavity and shell, respectively. UV light irradiation triggered the release of α-CD-RhB because of the trans-to-cis isomerization of the azobenzene residues in the capsule shell, whereas FITC-Dex was retained stably in the cavity because the microcapsules were stabilized by the host–guest complexation between β-CD-PAsp and adamantane. On the other hand, the microcapsules were slowly decomposed in tumor cells through hydrolysis of the PLGVR peptide linker by matrix metalloproteinase, which is over-expressed in tumor cells. Thus, the release of different drugs from the microcapsules could be independently controlled by photoirradiation and enzymes. Multi-drug delivery systems may be useful for chemotherapy treatment of cancer.

**Figure 13:**
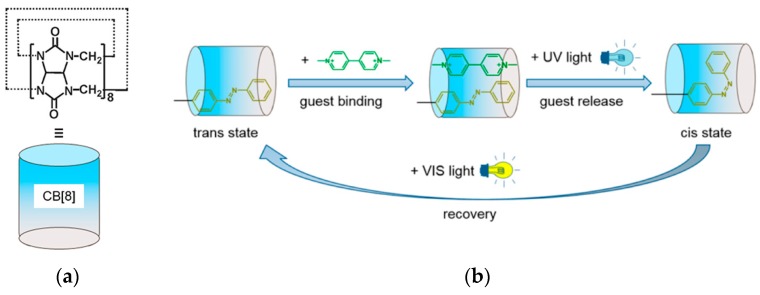
Chemical structure of cucurbit[8]uril (CB[8]) (**a**) and binding and release of methyl viologen (MV) in LbL film containing CB[8] (**b**). Reprinted with permission from Refs. [100,101]. Copyright 2015 and 2016 American Chemical Society.

Cucurbit[*n*]urils (CB[*n*]s) are a family of barrel-shaped synthetic compounds that exhibit extremely high affinity to positively-charged guest species [95,96]. Because of their strong affinity, CB[*n*] compounds have been used as components of LbL assemblies to bind and release guest molecules [97,98,99,100]. In this context, Schönhoff and coworkers prepared CB[8]-containing LbL films for photo-triggered binding and release of guest molecules (Figure 13) [101]. A quartz slide was alternately immersed in solutions of PAA and azobenzene polymers in the presence of CB[8] to form a LbL film, in which the azobenzene residues were included as guests in the cavity of CB[8]. The LbL film was subsequently immersed in a solution containing methyl viologen (MV). Spectroscopic studies showed that MV was accommodated into the CB[8] cavity as a second guest to form ternary host–guest complexes in the film. As illustrated in Figure 13, UV light irradiation expelled MV from the CB[8] cavity due to trans-to-cis isomerization of the azobenzene residues, resulting in the release of MV from the film. MV was repeatedly bound into the film and released by UV and visible light irradiation 10 times without degradation.

The examples discussed above in this section demonstrate the effectiveness of UV light irradiation in the controlled release of drug models from azobenzene-containing LbL films and microcapsules.

## 4. Conclusions

Azobenzene-containing LbL films and microcapsules have been prepared with a range of cationic and anionic polymers bearing azobenzene residues in their side chains or backbones. In most cases, azobenzene residues in the LbL assemblies exhibit trans-to-cis isomerization under UV light irradiation, followed by changes in the physical and chemical properties of the LbL assemblies. Therefore, azobenzene-containing LbL films and microcapsules have been studied for the construction of photosensitive devices. This review summarized the syntheses of azobenzene-containing LbL films and microcapsules and their biomedical applications including the photo-control of cell adhesion, the permeability of ion gates, and controlled release. Recent works have demonstrated the usefulness of azobenzene-containing LbL assemblies in the construction of photo-sensitive biomedical devices. An advantage of azobenzene-containing LbL assemblies is that the amount of azobenzene residues can be precisely controlled by changing the number of layers in the films, which enables regulation of the performance characteristics of the LbL assemblies. However, a drawback of LbL assemblies is their slow response to photoirradiation; in some cases, it takes several tens of minutes to reach a steady state under irradiation. For biomedical applications of these devices, a light source with longer wavelengths should be used because of the lower toxicity levels in cells and tissues. The appropriate design of azobenzene derivatives with conjugated systems may extend the absorption bands to a longer wavelength. Azobenzene-containing LbL films and microcapsules could be used in biomedical fields as photosensitive devices if these problems can be solved.
